# Book Reviews

**Published:** 1901-12

**Authors:** 


### BOOK REVIEWS.

NOTHNAGEL'S ENCYCLOPEDIA OF PRACTICAL MEDICINE. Edited by Alfred Stengel, M.D., Professor of Clinical Medicine in the University of Pennsylvania; Visiting Physician to the Pennsylvania Hospital.

It is universally acknowledged that the Germans lead the world in Internal Medicine; and of all the German works on this subject Nothnagel's "Encyclopedia of Special Pathology and Therapeutics" is conceded by scholars to be without question the best system of medicine in existence. So necessary is this book in the study of Internal Medicine that it comes largely to this country in the original German. In view of these facts, Messrs. W. B. Saunders & Company have arranged with the publishers to issue at once an authorized edition of this great encyclopedia of medicine in English.

For the present a set of some ten or twelve volumes, representing the most practical part of this encyclopedia, and selected with especial thought of the needs of the practical physician, will be published. The volumes will contain the real essence of the entire work, and the purchaser will therefore obtain at less than half the cost the cream of the original. Later the special and more strictly scientific volumes will be offered from time to time.

The work will be translated by men possessing thorough knowledge of both English and German.

Each volume will be edited by a prominent specialist on the subject to which it is devoted. It will thus be brought thoroughly up to date.

The American edition will be more than a mere translation of the German, for, in addition to the matter contained in the original, it will represent the very latest views of the leading American specialists in the various departments of Internal Medicine. The whole system will be under the editorial supervision of Dr. Alfred Stengel, who will select the subjects for the American edition, and will choose the editors of the different volumes.

Unlike most encyclopedias, the publication of this work will not be extended over a number of years, but five or six volumes will be issued during the coming year and the remainder of the series at the same rate. Moreover, each volume will be revised to the date of its publication by the American editor. This will obviate the objection that has heretofore existed to systems published in a number of volumes, since the subscriber will receive the completed work while the earlier volumes are still fresh.

The usual method of publishers, when issuing a work of this kind, has been to compel physicians to take the entire system. This seems to us in many cases to be undesirable. Therefore, in purchasing this encyclopedia *physicians will be given the opportunity of subscribing for the entire system at one time ; but any single volume or any number of volumes may be obtained by those who do not desire the complete series.* This latter method, while not so profitable to the publisher, offers to the purchaser many advantages which will be appreciated by those who do not care to subscribe for the entire work at one time.

This American edition of Nothnagel's Encyclopedia will, without question, form the greatest system of medicine ever produced, and the publishers feel confident that it will meet with general favor in the medical profession.

---

THE AMERICAN ILLUSTRATED MEDICAL DICTIONARY. For Practitioners and Students. A complete dictionary of the terms used in medicine, surgery, dentistry, pharmacy, chemistry, and the kindred branches, including much collateral information of an encyclopedic character, together with new and elaborate tables of arteries, muscles, nerves, viens, etc. ; of bacilli, bacteria, micrococci, streptococci ; Eponymic tables of diseases, operations, signs and symptoms, stains, tests, methods of treatment, etc., etc. By W. A. Newman Dorland, A.M., M.D., editor of the "American Pocket Medical Dictionary." Second edition, revised. Handsome large octavo, nearly 800 pages, bound in full flexible leather. Philadelphia and London : W. B. Saunders & Company, 1901. Price, $4.50 net.

A large first edition of the work was issued in October, 1900. From the day of its publication the book met with a remarkably large sale, and the edition was exhausted in eight months. This immediate success is doubtless due to certain special features which distinguish this work from other books of its kind. The avowed object of the author has been to furnish in a volume of convenient size an up-to-date dictionary, sufficiently full for the requirements of all classes of medical men; or, in other words, to give a maximum of matter in a minimum of space, and at the lowest possible cost. This object has been secured by the use of a large page, thin bible paper, and a flexible leather binding. The result is a truly luxurious specimen of bookmaking.

In this edition the book has been carefully revised. The author has also added upward of one hundred important new terms that have appeared in medical literature during the past few months. Among them appear "Anopheles," "Cryoscopy," "Johimbin," "Hemolysin," "Hedonal," "Sarectomy," etc., words that have recently come prominently before the profession, and which of course are not to be found in any other dictionary.

Other valuable features of the book are to be found in the complete and satisfactory definitions, the etymological references in the original languages, and the clear method of indicating the pronunciation. There are over one hundred new tables, and the illustrations add greatly to the usefulness of the book.

In the preface the author avows his intention of making the work represent as fully as possible the live literature of the medical sciences by keeping it in all respects thoroughly up to date.

---

THE DIAGNOSIS OF NERVOUS AND MENTAL DISEASES. By Howell T. Pershing, M.Sc., M.D., Professor of Nervous and Mental Diseases in the University of Denver; Neurologist to St. Luke's Hospital; Consultant in Nervous and Mental Diseases to the Arapahoe County Hospital; Member of the American Neurological Association. Illustrated. 12mo. Published by P. Blakiston's Son & Co., 1012 Walnut St., Philadelphia. 1901. Price, in cloth, $1.25 net.

This book is intended to aid the general practitioner in making a diagnosis of nervous and mental disease. Imitating the method in vogue of teaching botany, the author has arranged a series of tables which furnish the "key" to the recognition of the disease. It is not designed to replace the larger text books on neurology. Localization and the complex system of reasoning from this to the disease is made very much less laborious by these carefully arranged synopses, which very fully cover the field. The worth of the book will be apparent at once, and there can be no doubt but that it will confer much benefit upon the general practitioner.

---

A TREATISE ON SURGERY BY AMERICAN AUTHORS.—For Students and Practitioners of Medicine and Surgery. Edited by Roswell Park, M.D., Professor of Surgery in the University of Buffalo, N. Y. New (3d) Edition in one Royal Octavo Volume of 1350 pages with 692 engravings and 64 full-page plates in colors and monochrome. Cloth, $7.00, *net;* leather, $8.00, *net.* Lea Brothers & Co., Philadelphia and New York.

The new edition of Park's Surgery is the third in five years, which attests its popularity and the great activity in surgery manifested here and elsewhere. The best special knowledge is collected on each subject so that it will serve equally well the advanced student of medicine and the practitioner. New illustrations of a high order of excellence have been added, which brings the number up to seven hundred pictures and sixty-four full-page plates in colors and monochrome. The work is thoroughly revised and up to date.

---

THE PHYSICIAN'S POCKET ACCOUNT BOOK.

This handy book is intended to be carried in the breast-pocket. It is very complete and compact and will be found very useful for the busy doctor and will probably save him many a dollar which would otherwise be forgotten and lost. It has an index, balances due, pages for entering account of professional services rendered, columns ruled for obstetrical cases and vaccination records, and ample space for entering cash receipts. The book is bound in black leather and presents a very handsome appearance. It is issued by the Medical Council, 12th and Walnut Sts., Philadelphia.

